# Supplementary material for: Theoretical and experimental analysis of circularly polarized luminescence spectrophotometers for artifact-free measurements using a single CCD camera
Source: Nat Commun. 2023 Feb 24;14:1065. doi: 10.1038/s41467-023-36782-9 (PMC9958114; doi:10.1038/s41467-023-36782-9)
Supplement: Supplementary file 1 — Supplementary information [file 41467_2023_36782_MOESM1_ESM.pdf]

Supplementary Information for Theoretical and experimental analysis of circularly polarized luminescence spectrophotometers for artifact-free measurements using a single CCD camera

B. Baguenard[1], A. Bensalah-Ledoux[1], L. Guy[2], F. Riobé[2] , O. Maury[2] and S. Guy[1]

[1] Univ Lyon, Univ Claude Bernard Lyon 1, CNRS, Institut Lumière Matière, F-69622, VILLEURBANNE, France

[2] Univ Lyon, ENS de Lyon, CNRS UMR 5182 Laboratoire de Chimie, F-69342 Lyon, France

Contents

1 Description of home made imaging spectrometer 2

2 Optical Components 2

3 Cross-talk 3

4 ±45° (LD') and circular dichroisms (CD) of the polarizing beam spliter 4

5 Order of magnitudes 5

6 Mueller matrices analyses 5

6.1 Emitted Stokes vector 5
6.2 Effect of the quarter waveplate 6
6.3 Polarizing BeamSplitter (PBS) 7
6.4 Wavelength mismatch 7
6.5 Stokes vector on the camera 7

7 Polarized intensity combinations 8

7.1 Spatial separation of the polarization 8
7.2 Temporal separation of the polarization 8
7.3 Spatial and temporal separation of the polarization 9

8 Luminescence theoretical results 9

9 Tilted images 9

10 IR CPL of $Yb^{3}+$ complexes 10

11 Linearity and SNR 11

12 Mixing linear and PBS defects through fluorescein 12

13 Chemical 12

14 Photoproperties 13

1 Description of home made imaging spectrometer

The images of the fibers outputs are made on the entrance slit of the home-made spectrometer, schematized in Supplementary Figure 1, thanks to a triplet lens (L_1). The entrance slit is then imaged onto the CCD camera by means of two achromatic lenses ($L_{2,3}$) with a transmission grating from ThorLabs, in between. By playing with the grating and the focal lengths of $L_{2,3}$, we adjust the wavelength range depending on the studied molecules. The two CPL spectra are thus, recorded on the CCD.

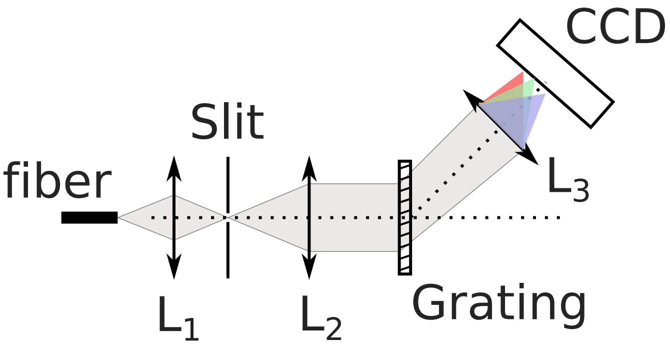

Supplementary Figure 1: Schematic of the home-made spectrometer top view. L_1: triplet achromatic lens (f=40mm). $L_{2,3}$ achromatic doublet.

2 Optical Components

**Component name**	**Manufacturer**	**Part Number**	**Specifications**
Quarter Wave plate	Thor Labs	AQWP05M-600	$\frac{\lambda}{4} \pm 0.01\lambda$
Polarizing beam spliter	Thor Labs	PBS-251	420-680 nm, TP/TS> 100
Polarizing beam spliter	Thor Labs	PBS-253	0.9-1.3 µm, TP/TS>100
Grating	Thor Labs	GT25-08	830 gr/mm VIS
Grating	Thor Labs	GTI25-03	300 gr/mm IR
Fiber bundle	IDIL		200 µm, 300-1200 nm
CCD Camera	Andor	Idus 420	16bits, 1024x256 pixels

Supplementary Table 1: List of optical parts used in this work with manufacturer, part numbers and relevant specifications

3 Cross-talk

The mixing between the two polarization-encoded channels was measured as follows. The light beam from a white lamp is collimated onto the PBS. A Glan polarizer is inserted between the lamp and the PBS. The transmitted and reflected signals are recorded for the horizontally or vertically polarized incident beam. The blue and red curves in Supplementary Figure 2 show the ratio between the blocked and unblocked polarizations.

The straylight induces also crosstalk between the two arms. It is measure by sending light only in one arm and measuring the corresponding signal on the other one's (black curve in Supplementary Figure 2)

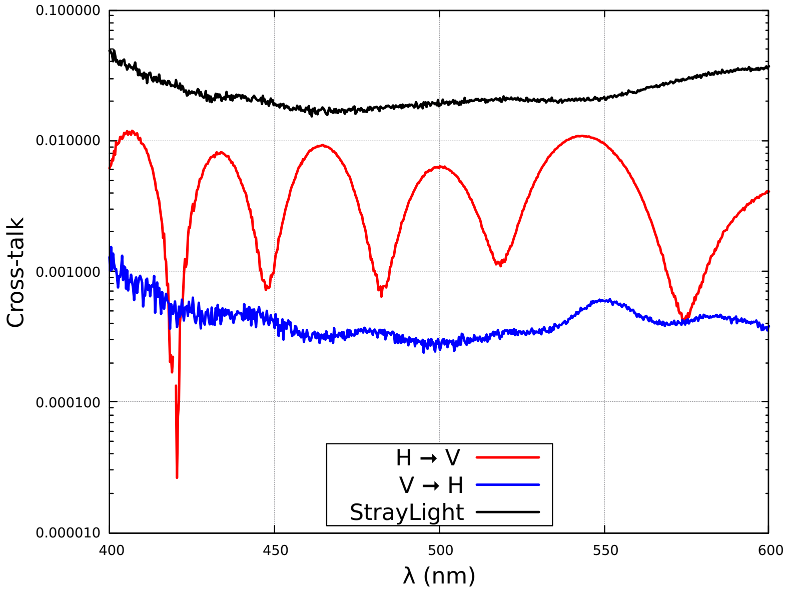

Supplementary Figure 2: Cross talk between the two arms. H→V curve is the ratio of the horizontally polarized light reflected by the PBS over the transmitted one's. V→H curve is the ratio of the vertically polarized light transmitted by the PBS over the reflected one's. The straylight is the measured signal on the CCD camera from one track corresponding to a given fiber but this fiber is not shined while the other one is.

4 ±45° (LD') and circular dichroisms (CD) of the polarizing beam spliter

The LD' and CD of the PBS were measured by placing the PBS into our home made LD/CD spectrometer based on a PEM modulator. The circular and linear dicroïsms are measured at the first and second harmonic respectively. Supplementary Figure 3 displays the results for the linear (LD'- top panels) and the circular dichroism (CD- bottom panels). First, second and third rows corresponds to the transmitted beam, the reflected beam and the differential response between the two beams respectively. Because the LD' values are in the order of magitude of the CD one's we are sure that this CD does not come from experimental artifacts.

We found that a few percents residual LD' and CD are present in the PBS. Moreover, the CD value is dependent on what part of the PBS is used: we scanned the PBS from one side to the other one's by step of 2 mm and recorded CD variations of more than 2%.

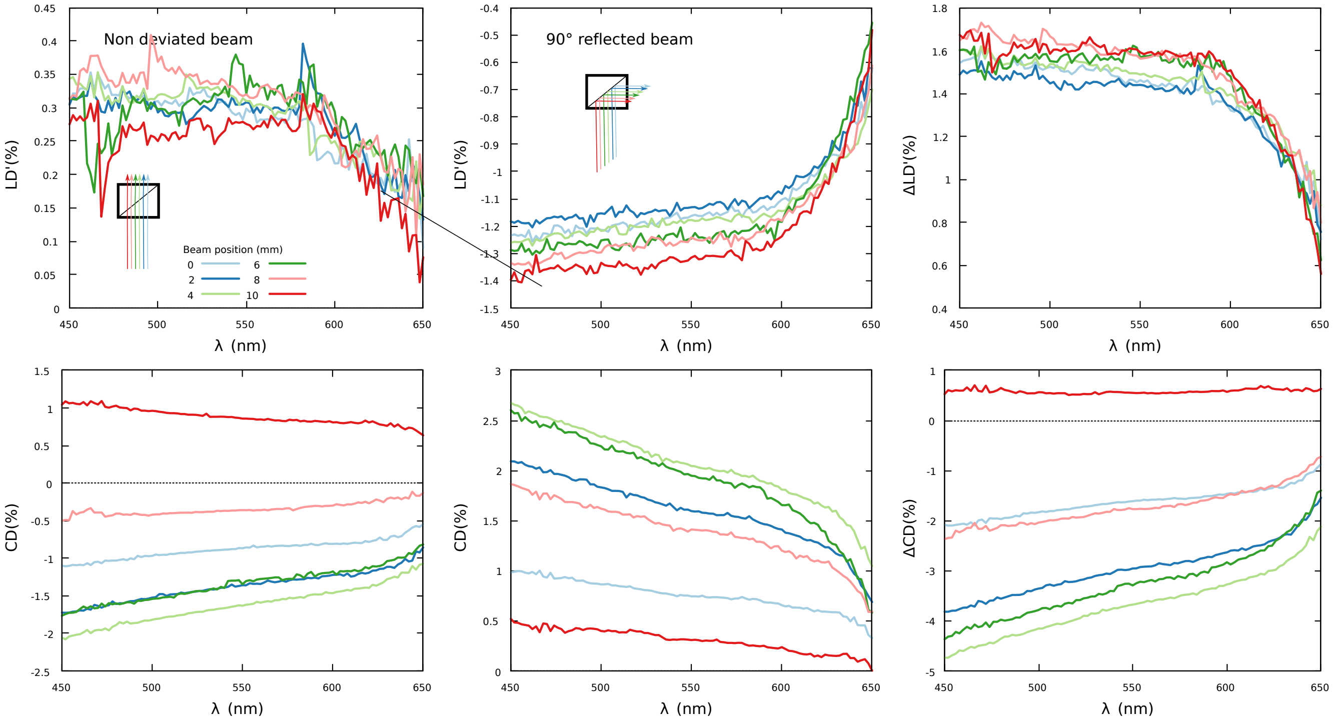

Supplementary Figure 3: Measured LD' and CD dichroims in our CD/LD spectrometers. The probing beam comes from a white lamp spectrally resolved via a monochromator. The image of the beam on the PBS is a 2x10 mm rectangle. The response of the PBS was probed by longitudinally scanning its position by steps of 2mm. The last colum is the difference between the two first ones.

5 Order of magnitudes

Spectroscopic signals S_0 is the zero order parameter. We assume here that we are working on small dichroic spectra. Therefore, $S_0 \gg S_1,\ S_2,\ S_3$

Experimental parameters All the parameters related to instrumental defects are dimensionless. They are much smaller than one: $LD'_p,\ CD_p, \epsilon_\pm,\ \Delta\phi,\ \Delta T,\ \alpha$ and $\psi \ll 1$

Derivative signals Our calculations put into evidence signal distortion coming from derivative signals times the wavelength mismatch between the two spectroscopic channels. The order of magnitude can be estimated according to:

$$\Delta\lambda \frac{\partial S_i}{\partial \lambda} \approx \Delta\lambda \frac{S_i}{FWHM} = \frac{\Delta\lambda}{FWHM} S_i \tag{1}$$

We assume here that $\frac{\Delta\lambda}{FWHM} \ll 1$.

6 Mueller matrices analyses

To demonstrate the accuracy of our method, using quarterwave plate (QWP) rotation, we use the Stokes-Mueller formalism. Light is described using a 4-component vector, which are

- S_0 : total intensity, equal to $I_{LCP} + I_{RCP}$ (sum of Left and Right-handed Circular Polarizations);
- S_1 and S_2 : differences of linear polarized intensities; $I_{0°} - I_{90°}$ for S_1, $I_{45°} - I_{-45°}$ for S_2. References axes (0° and 90°) are choosen identical to the PBS ones. These parameters are equal to 0 in a solution.
- $S_3 = I_{RCP} - I_{LCP}$, opposite to the CPL.

Identification of the configurations: the p and q parameters In the following, p=±1 denotes the polarization channel after the PBS (+ and - fort the horizontal and vertical polarization respectively) and q=±1 denotes the orientation of the QWP fast axis (± for $\pm\pi/4$). The parameter p is therefore affected to (i) the polarization channel ($\mathbf{P}_p$), (ii) the transmitted intensity according to the two pathes ($\mathbf{T}_p$) and (iii) the measure wavelength λ_p. The parameter q describes both the QWP orientation $\mathbf{R}(\pm 2q\theta)$ and the pump intensity corresponding to these two orientations ϕ_q.

6.1 Emitted Stokes vector

The intensity of the pump source changes the amplitude of the initial Stokes vector. We call $\phi_{q=1}$ and $\phi_{q=-1}$ the excitation ratio for the first measurement (QWP azimuth at $+\pi/4$) and the second one's (QWP azimuth at $-\pi/4$) respectively. The emitted Stokes vector is:

$$\mathbf{S}^e_q = \phi_q \mathbf{S}^e \tag{2}$$

We define the average pump excitation $\tilde{\phi}$ and the relative difference:

$$\tilde{\phi} = \frac{\phi_{+1} + \phi_{-1}}{2}, \quad \Delta\phi = \frac{\phi_{+1} - \phi_{-1}}{\phi_{+1} + \phi_{-1}} \tag{3}$$

From these definitions:

$$\phi_q = \tilde{\phi}\,(1 + q\Delta\phi) \tag{4}$$

With all these definitions, the initial emitted light is written as follow

$$\mathbf{S}^e_q = \tilde{\phi}(1 + q\Delta\phi) \begin{pmatrix} S^e_0 \\ S^e_1 \\ S^e_2 \\ S^e_3 \end{pmatrix} \tag{5}$$

6.2 Effect of the quarter waveplate

The Mueller matrix of the QWP is written in the laboratory frame. Its retardance is $\Psi = \pi/2 + \psi$. ψ is wavelength dependent.

$$\mathbf{Q} = \begin{pmatrix} 1 & 0 & 0 & 0 \\ 0 & 1 & 0 & 0 \\ 0 & 0 & \cos\Psi & -\sin\Psi \\ 0 & 0 & \sin\Psi & \cos\Psi \end{pmatrix} \approx \begin{pmatrix} 1 & 0 & 0 & 0 \\ 0 & 1 & 0 & 0 \\ 0 & 0 & -\psi & -1 \\ 0 & 0 & 1 & -\psi \end{pmatrix} \tag{6}$$

at first order in ψ.

The azimuth rotation angle $\theta_\mathrm{q} = q\frac{\pi}{4} + \alpha$ where $q = \pm 1$. α is the error on the QWP orientation, supposed to be small ($\alpha \ll \pi$). The Mueller matrix of the rotated QWP is deduced from the one written in the laboratory frame by using the rotator:

$$\mathbf{R}(2\theta_\mathrm{q}) = \begin{pmatrix} 1 & 0 & 0 & 0 \\ 0 & \cos 2\theta_\mathrm{q} & -\sin 2\theta_\mathrm{q} & 0 \\ 0 & \sin 2\theta_\mathrm{q} & \cos 2\theta_\mathrm{q} & 0 \\ 0 & 0 & 0 & 1 \end{pmatrix} \approx \begin{pmatrix} 1 & 0 & 0 & 0 \\ 0 & -2\mathrm{q}\alpha & -\mathrm{q} & 0 \\ 0 & \mathrm{q} & -2\mathrm{q}\alpha & 0 \\ 0 & 0 & 0 & 1 \end{pmatrix} \tag{7}$$

$$\mathbf{R}^{-1}(2\theta_\mathrm{q}) = \begin{pmatrix} 1 & 0 & 0 & 0 \\ 0 & \cos 2\theta_\mathrm{q} & \sin 2\theta_\mathrm{q} & 0 \\ 0 & -\sin 2\theta_\mathrm{q} & \cos 2\theta_\mathrm{q} & 0 \\ 0 & 0 & 0 & 1 \end{pmatrix} \approx \begin{pmatrix} 1 & 0 & 0 & 0 \\ 0 & -2\mathrm{q}\alpha & \mathrm{q} & 0 \\ 0 & -\mathrm{q} & -2\mathrm{q}\alpha & 0 \\ 0 & 0 & 0 & 1 \end{pmatrix} \tag{8}$$

Mueller matrix of the rotated QWP The Mueller matrice of the WQP is found via:

$$\mathrm{q}_\mathrm{q} = \mathbf{R}^{-1}(2\theta_\mathrm{q})\mathrm{q}\mathbf{R}(2\theta_\mathrm{q})$$

At first order we get:

$$\mathrm{q}_\mathrm{q} = \begin{pmatrix} 1 & 0 & 0 & 0 \\ 0 & -\psi & 2\alpha & -\mathrm{q} \\ 0 & 2\alpha & 1 & 2\mathrm{q}\alpha \\ 0 & \mathrm{q} & -2\mathrm{q}\alpha & -\psi \end{pmatrix} \tag{9}$$

Stokes vector after the QWP The Stokes vector after the QWP writes:

$$\mathbf{S}^{\mathbf{qwp}}_\mathrm{q} = \mathbf{Q}_\mathrm{q}\mathbf{S}^{e}_\mathrm{q} = \tilde{\phi}(1 + \mathrm{q}\Delta\phi) \begin{pmatrix} \mathrm{S}^\mathrm{e}_0 \\ -\mathrm{q}\mathrm{S}^\mathrm{e}_3 \\ \mathrm{S}^\mathrm{e}_2 \\ \mathrm{q}\mathrm{S}^\mathrm{e}_1 \end{pmatrix} + \tilde{\phi}(1 + \mathrm{q}\Delta\phi) \begin{pmatrix} 0 \\ -\psi\mathrm{S}^\mathrm{e}_1 + 2\alpha\mathrm{S}^\mathrm{e}_2 \\ 2\alpha\mathrm{S}^\mathrm{e}_1 + 2\mathrm{q}\alpha\mathrm{S}^\mathrm{e}_3 \\ -2\mathrm{q}\alpha\mathrm{S}^\mathrm{e}_2 - \psi\mathrm{S}^\mathrm{e}_3 \end{pmatrix}$$

The first right hand side Stokes vector corresponds to the ideal QWP oriented at ±45° for which:

- the circular polarizations are converted to horizontal–vertical linear polarizations ($S^{qwp}_1 = \pm\mathrm{S}^\mathrm{e}_3$)
- the ±45° linear polarization are aligned along the main axes and are therefore unaffected by the device ($S^{qwp}_2 = \mathrm{S}^\mathrm{e}_2$)
- the horizontal–vertical polarizations are converted to left–right circular polarizations ($S^{qwp}_3 = \pm\mathrm{S}^\mathrm{e}_1$)

This expression may be written:

$$\mathbf{S}^{qwp}_\mathrm{q} = \mathbf{S}^{qwp_0}_\mathrm{q} + \Delta\mathbf{S}^{pump}_\mathrm{q} + \Delta\mathbf{S}^{qwp}_\mathrm{q} \tag{10}$$

where $\mathbf{S}^{qwp_0}_\mathrm{q}$, $\Delta\mathbf{S}^{pump}_\mathrm{q}$ and $\Delta\mathbf{S}^{qwp}_\mathrm{q}$ are the ideal, first order pump and QWP correction Stokes vectors respectively defined as:

$$\mathbf{S}^{qwp_0}_\mathrm{q} = \tilde{\phi} \begin{pmatrix} \mathrm{S}^\mathrm{e}_0 \\ -\mathrm{q}\mathrm{S}^\mathrm{e}_3 \\ \mathrm{S}^\mathrm{e}_2 \\ \mathrm{q}\mathrm{S}^\mathrm{e}_1 \end{pmatrix}, \quad \Delta\mathbf{S}^{pump}_\mathrm{q} = \mathrm{q}\Delta\phi\mathbf{S}^{qwp_0}_\mathrm{q}, \quad \Delta\mathbf{S}^{qwp}_\mathrm{q} = \tilde{\phi} \begin{pmatrix} 0 \\ -\psi\mathrm{S}^\mathrm{e}_1 + 2\alpha\mathrm{S}^\mathrm{e}_2 \\ 2\alpha\mathrm{S}^\mathrm{e}_1 + 2\alpha\mathrm{q}\mathrm{S}^\mathrm{e}_3 \\ -2\mathrm{q}\alpha\mathrm{S}^\mathrm{e}_2 - \psi\mathrm{S}^\mathrm{e}_3 \end{pmatrix} \tag{11}$$

6.3 Polarizing BeamSplitter (PBS)

Main axes are chosen horizontal and vertical. The p underscript denotes the horizontal (+1) or vertical (-1) transmission. We note $\epsilon_{\rm p}$ the imperfection of the polarization splitting set-up taking into account the PBS extinction ratio and the stray light. Both limitations mix one polarization in the other one's. Transmission is one along one axis and $\epsilon_{\rm p} \ll 1$ along the orthogonal axis. We also take into account the $\pm 45°$ and circular dicroism of the PBS as $\mathrm{LD'_p} \ll 1$ and $\mathrm{CD_p} \ll 1$ respectively. The corresponding Mueller matrix is therefore (only the first line is relevant here) :

$$\mathbf{P}_{\rm p} = \frac{1}{2}\begin{pmatrix} 1 & \mathrm{p}(1-\epsilon_{\rm p}) & \mathrm{LD'_p} & \mathrm{CD_p} \\ \cdot & \cdot & \cdot & \cdot \\ \cdot & \cdot & \cdot & \cdot \\ \cdot & \cdot & \cdot & \cdot \end{pmatrix} \tag{12}$$

It can also be written as the sum of a zero and first order matrices:

$$\mathbf{P}_{\rm p} = \mathbf{P}^0_{\rm p} + \Delta\mathbf{P}_{\rm p} \tag{13}$$

where the zero and first order matrices are :

$$\mathbf{P}^0_{\rm p} = \frac{1}{2}\begin{pmatrix} 1 & \mathrm{p} & 0 & 0 \\ \cdot & \cdot & \cdot & \cdot \\ \cdot & \cdot & \cdot & \cdot \\ \cdot & \cdot & \cdot & \cdot \end{pmatrix}, \quad \Delta\mathbf{P}_{\rm p} = \frac{1}{2}\begin{pmatrix} 0 & -\mathrm{p}\epsilon_{\rm p} & \mathrm{LD'_p} & \mathrm{CD_p} \\ \cdot & \cdot & \cdot & \cdot \\ \cdot & \cdot & \cdot & \cdot \\ \cdot & \cdot & \cdot & \cdot \end{pmatrix} \tag{14}$$

Transmission of the optical path All the optical element have their own transmission. We note $\mathrm{T}_{p=\pm1}$ the transmission of the horizontally and vertically polarized channels respectively. The corresponding Mueller matrix are:

$$\mathbf{T}_{\rm p} = \mathrm{T_p}\mathbf{I}$$

where $\mathbf{I}$ is the identity matrix. We define the average transmission $\tilde{T}$ and the relative transmission difference:

$$\tilde{T} = \frac{\mathrm{T}_{+1} + \mathrm{T}_{-1}}{2}, \quad \Delta\mathrm{T} = \frac{\mathrm{T}_{+1} - \mathrm{T}_{-1}}{\mathrm{T}_{+1} + \mathrm{T}_{-1}} \tag{15}$$

Fron these definitions:

$$T_{\rm p} = \tilde{T}(1 + \mathrm{p}\Delta\mathrm{T}) \tag{16}$$

6.4 Wavelength mismatch

The light collected on the top and bottom tracks on the camera are horizontally dispersed in wavelength along the columns of the CCD matrix. However, we can not be sure that the wavelength is the same all over the columns. Main origin of this mismatch is the imperfect alignment between the entrance slit, the grating and the CCD camera. Therefore the recorded spectra have different wavelength dependencies: $\mathbf{S}(\lambda_{p=\pm1})$ for the horizontally and vertically polarized channels respectively.

By writing $\Delta\lambda = \frac{\lambda_{+1}-\lambda_{-1}}{2}$ and $\lambda = \frac{\lambda_{+1}+\lambda_{-1}}{2}$, we get at the first order:

$$\mathbf{S}(\lambda_{\rm p}) = \mathbf{S}(\lambda + \mathrm{p}\Delta\lambda) \approx \mathbf{S}(\lambda) + \mathrm{p}\Delta\lambda\frac{\partial\mathbf{S}}{\partial\lambda}(\lambda) \tag{17}$$

6.5 Stokes vector on the camera

Taking all the parameters described above, the four Stokes vectors recorded at the camera are

$$\mathbf{S}^{cam}_{\rm pq} = \mathbf{T}_{\rm p}\mathbf{P}_{\rm p}\mathbf{S}^{qwp}_{\rm q}$$

By writing explicitly the zero and first order terms for the PBS (eq. 14) and $\mathbf{S}^{qwp}_{\rm q}$ (eq. 11), we get:

$$\mathbf{S}^{cam}_{pq} = \tilde{T}(1+\mathrm{p}\Delta\mathrm{T})\left(\mathbf{P}^0_{\rm p} + \Delta\mathbf{P}_{\rm p}\right)\left(\mathbf{S}^{\rm qwpo}_{\rm q} + \Delta\mathbf{S}^{\rm pump}_{\rm q} + \Delta\mathbf{S}^{\rm qwp}_{\rm q}\right)(\lambda_{\rm p})$$

At first order :

$$\frac{1}{\tilde{T}}\mathbf{S}^{cam}_{pq} = \underbrace{\mathbf{P}^0_{\rm p}\mathbf{S}^{qwpo}_{\rm q}}_{\text{zero order}} + \underbrace{\mathrm{p}\Delta\mathrm{T}\mathbf{P}^0_{\rm p}\mathbf{S}^{qwpo}_{\rm q}}_{\text{mis-balance}} + \underbrace{\mathbf{P}^0_{\rm p}\Delta\mathbf{S}^{pump}_{\rm q}}_{\text{pump imperfections}} + \underbrace{\Delta\mathbf{P}_{\rm p}\mathbf{S}^{qwpo}_{\rm q}}_{\text{PBS imperfections}} + \underbrace{\mathbf{P}^0_{\rm p}\Delta\mathbf{S}^{qwp}_{\rm q}}_{\text{QWP imperfections}} \tag{18}$$

The first term, as the zero order, is the ideal output Stokes vector without any imperfection. The two following terms takes into account the "scalar" departure from the ideal case via the spatial and temporal mis-balance of the set-up. These two contributions are proportional to the zero order terms and can be treated as a response function. The two last terms take into account the imperfection of the polarizer and the QWP. These last terms are no more proportional to the ideal output Stokes vector and therefore can not be treated as a response function. Using the expression of $\Delta\mathbf{S}_{\mathrm{q}}^{pump}$, this equation rewrites:

$$\frac{1}{\tilde{T}}\mathbf{S}_{pq}^{cam} = \mathbf{P}_{\mathrm{p}}^{0}\mathbf{S}_{\mathrm{q}}^{qwp_0}(1+\mathrm{p}\Delta\mathrm{T}+\mathrm{q}\Delta\phi)+\Delta\mathbf{P}_{\mathrm{p}}\mathbf{S}_{\mathrm{q}}^{qwp_0}+\mathbf{P}_{\mathrm{p}}^{0}\Delta\mathbf{S}_{\mathrm{q}}^{qwp}$$

The measured intensities $\mathrm{I_{pq}}$ at the both channels are the first component of the $\mathbf{S}_{\mathrm{pq}}^{cam}$ Stokes vectors :

$$\frac{2}{\tilde{T}\tilde{\phi}}\mathrm{I_{pq}} = (1+\mathrm{p}\Delta\mathrm{T}+\mathrm{q}\Delta\phi)\left[\mathrm{S_0^e}-\mathrm{pqS_3^e}\right](\lambda_{\mathrm{p}})+\left[\mathrm{pq}\epsilon_{\mathrm{p}}\mathrm{S_3^e}+\mathrm{LD'_pS_2^e}+\mathrm{qCD_pS_1^e}\right](\lambda_{\mathrm{p}})+\left[-\mathrm{p}\psi\mathrm{S_1^e}+2\mathrm{p}\alpha\mathrm{S_2^e}\right](\lambda_{\mathrm{p}}) \quad (19)$$

It simplifies at first order according to :

$$\begin{aligned}\frac{2}{\tilde{T}\tilde{\phi}}\mathrm{I_{pq}}(\lambda) =&(1+\mathrm{p}\Delta\mathrm{T}+\mathrm{q}\Delta\phi)\mathrm{S_0^e}+\mathrm{p}\Delta\lambda\frac{\partial\mathrm{S_0^e}}{\partial\lambda}+(-\mathrm{p}\psi+\mathrm{qCD_p})\mathrm{S_1^e}\\&+(\mathrm{LD'_p}+2\mathrm{p}\alpha)\mathrm{S_2^e}-(\mathrm{pq}+\mathrm{q}\Delta\mathrm{T}+\mathrm{p}\Delta\phi-\mathrm{pq}\epsilon_{\mathrm{p}})\mathrm{S_3^e}-\mathrm{q}\Delta\lambda\frac{\partial\mathrm{S_3^e}}{\partial\lambda} \quad (20)\end{aligned}$$

In this expression we code in red, blue and green the terms dependent on the arm path only, the QWP orientation only and both path and QWP respectively.

7 Polarized intensity combinations

7.1 Spatial separation of the polarization

Here, we add and subtract the two signals coming from the two polarized encoded paths ($p=\pm1$) but with the same QWP orientation (q maintained). The sum signal performs an average over the two arms, therefore the red and green terms in Equation 20 proportional to p cancel two by two leading to the average value. However, the $\epsilon_\pm$ being not antisymetric in respect to the arm (top panel of Supplementary Figure 3), it does not vanish by this averaging. For the difference signal, the p term are revealed and the $q=\pm1$ blue terms corresponding to the time fluctuations cancel each others:

$$\frac{1}{2\tilde{T}\tilde{\phi}}(\mathrm{I_{+1,q}}+\mathrm{I_{-1,q}}) =(1+\mathrm{q}\Delta\phi)\mathrm{S_0^e}+\mathrm{q}\widetilde{\mathrm{CD}}\mathrm{S_1^e}+\widetilde{\mathrm{LD}}'\mathrm{S_2^e}-\mathrm{q}(\Delta\mathrm{T}-\Delta\epsilon)\mathrm{S_3^e}-\mathrm{q}\Delta\lambda\frac{\partial\mathrm{S_3^e}}{\partial\lambda} \quad (21)$$

$$\frac{1}{2\tilde{T}\tilde{\phi}}(\mathrm{I_{+1,q}}-\mathrm{I_{-1,q}}) =\Delta\mathrm{TS_0^e}+\Delta\lambda\frac{\partial\mathrm{S_0^e}}{\partial\lambda}+(-\psi+\mathrm{q}\Delta\mathrm{CD})\mathrm{S_1^e}+(\Delta\mathrm{LD'}+2\alpha)\mathrm{S_2^e}-\mathrm{q}(1+\mathrm{q}\Delta\phi-\tilde{\epsilon})\mathrm{S_3^e} \quad (22)$$

where $\widetilde{LD}'=\frac{\mathrm{LD'_+}+\mathrm{LD'_-}}{2}$, $\widetilde{CD}=\frac{\mathrm{CD_+}+\mathrm{CD_-}}{2}$, $\tilde{\epsilon}=\frac{\epsilon_++\epsilon_-}{2}$, $\Delta\mathrm{LD'}=\frac{\mathrm{LD'_+}-\mathrm{LD'_-}}{2}$, $\Delta\mathrm{CD}=\frac{\mathrm{CD_+}-\mathrm{CD_-}}{2}$ and $\Delta\epsilon=\frac{\epsilon_+-\epsilon_-}{2}$.
The pre-factor "$1+q\Delta\phi$" for the $\mathrm{S_0^e}$ and $\mathrm{S_3^e}$ Stokes components corresponds to the intensity at the time of measurement. When just dealing with spatial separation, it can be replaced by 1.

7.2 Temporal separation of the polarization

Here, two measurements are performed on the same arm (p maintained) at two different times to invert the QWP ($q=\pm1$). Here, the "q" terms (blue and green in Equation. 20, time variation) vanishes by averaging and are kept for the subtraction. The red terms, specific to one arm, cancel after the subtraction. From Equation 20:

$$\frac{1}{2\tilde{T}\tilde{\phi}}(\mathrm{I_{p,1}}+\mathrm{I_{p,-1}}) =(1+\mathrm{p}\Delta\mathrm{T})\mathrm{S_0^e}+\mathrm{p}\Delta\lambda\frac{\partial\mathrm{S_0^e}}{\partial\lambda}-\mathrm{p}\psi\mathrm{S_1^e}+(\mathrm{LD'_p}+2\mathrm{p}\alpha)\mathrm{S_2^e}-\mathrm{p}\Delta\phi\mathrm{S_3^e} \quad (23)$$

$$\frac{1}{2\tilde{T}\tilde{\phi}}(\mathrm{I_{p,+1}}-\mathrm{I_{p,-1}}) =\Delta\phi\mathrm{S_0^e}+\mathrm{CD_pS_1^e}-\mathrm{p}(1+\mathrm{p}\Delta\mathrm{T}-\epsilon_{\mathrm{p}})\mathrm{S_3^e}-\Delta\lambda\frac{\partial\mathrm{S_3^e}}{\partial\lambda} \quad (24)$$

The pre-factor "1+pΔT" for the $\mathrm{S_0^e}$ and $\mathrm{S_3^e}$ Stokes components corresponds to the transmitted intensity of the arm under consideration. When just dealing with time separation, it can be replaced by 1. The differential term $\Delta\lambda\frac{\partial}{\partial\lambda}$ appears here because of the first order calculation. They correspond to the measurement at λ_{p} and they can be omitted when dealing with time measurements only.

7.3 Spatial and temporal separation of the polarization

Here we combine the four measurements. Calculation can be performed straight from Equation 20. The average fluorescence over the four measurement can also be calculated by averaging Eq.21 with q=±1 or Eq.23 with p=±1. In a same way, the CPL may be obtained straight from the I_{pq} expression or by subtract Eq. 22 with q=±1 or Eq. 24 with p=±1. The result is :

$$\frac{(I_{1,1} + I_{-1,1} + I_{1,-1} + I_{-1,-1})}{4\tilde{T}\tilde{\phi}} = S_0^e + \widetilde{LD'}S_2^e \tag{25}$$

$$\frac{(I_{1,1} - I_{-1,1}) - (I_{1,-1} - I_{-1,-1})}{4\tilde{T}\tilde{\phi}} = -(1-\tilde{\epsilon})S_3^e + \Delta CDS_1^e \tag{26}$$

8 Luminescence theoretical results

Luminescence is calculated at the first order for three signal combinations. ZO is the result at zero order. First order terms are in the fourth column. The last column shows the corresponding unwanted signals present in the main signal.

Polarization separation	Signals combination	ZO	1st Order	False Lum. signals
QWP rotation	$I_{p,+1} + I_{p,-1}$	$S_0(\lambda)$	$-p\Delta\phi \cdot S_3^e$<br>$-p\psi S_1^e + (2p\alpha +$<br>$LD'_p)S_2^e$	CPL<br>Linear Lum.
Two optical paths	$I_{+1,q} + I_{-,1q}$	$S_0(\lambda)$	$-q\Delta T\ S_3$<br>$-q\Delta\lambda\frac{\partial S_3}{\partial\lambda}$<br>$q\widetilde{CD}S_1^e + \widetilde{LD'}S_2^e$	CPL<br>CPL derivative<br>Linear Lum.
Four signals	$\sum_{p,q} I_{p,q}$	$S_0(\lambda)$	$\widetilde{LD'}S_2^e$	Linear Lum.

Supplementary Table 2: Luminescence calculated at different orders of the experimental limitations using Equation 20 for three signal combinations. $S^e_{i=0..3}$ are the Stokes components of the emitted light. The p and q subscript denotes the polarization channel and the QWP orientation respectively. ZO is the result at zero order.The first non null order terms are in the fourth column. The last column shows the corresponding unwanted spectral signals present in the main signal.

9 Tilted images

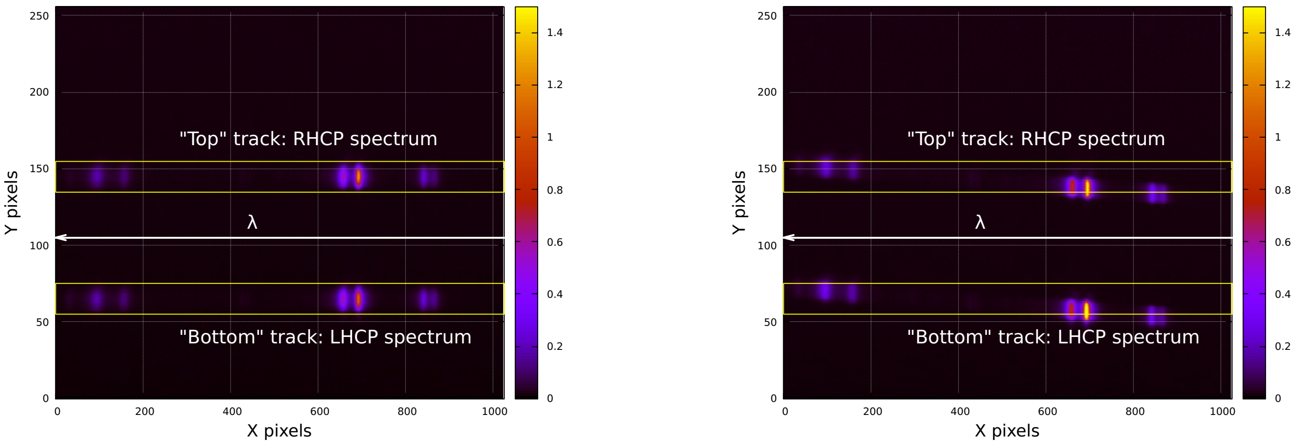

Supplementary Figure 4: Images on the camera recorded for Eu^{3+} complexes with best aligned (left) and mis-aligned (right) setup.

10 IR CPL of Yb^{3+} complexes

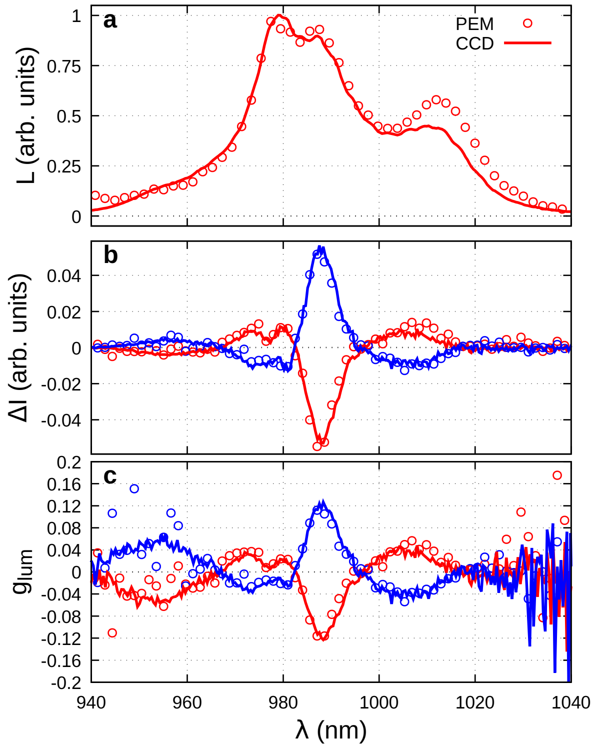

Supplementary Figure 5: CPL of Yb^{3+} complexes Luminescence (top), CPL (middle) and g_{lum} (bottom) for the two enantiomers of **3** under 405 nm excitation. The luminescence and CPL are normalized to the maximum of emission, so that the g_{lum} value at maximum luminescence, can be directly read on the y-axis. In continuous line spectra recorded with the CCD camera using the spatial-time procedure, in dashed line the spectra recorded in a step by step mode using a PEM+analyzer system. Wavelength response correction has been applied for each system.

11 Linearity and SNR

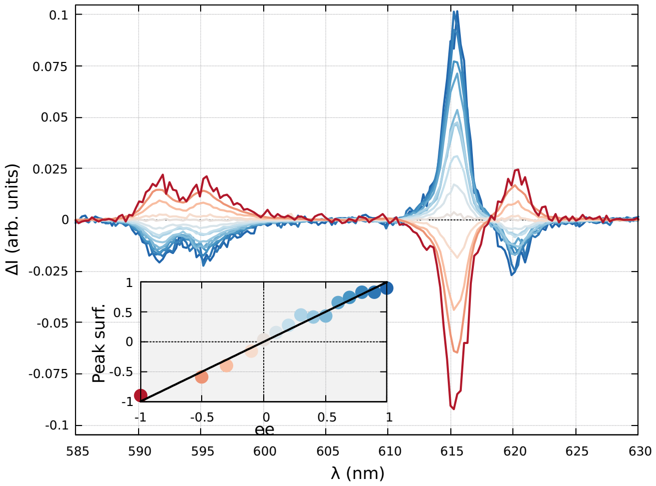

Supplementary Figure 6: CPL spectra measured for different enantiomeric excess ee. The inset displays the surface of the 615 nm peak versus ee.

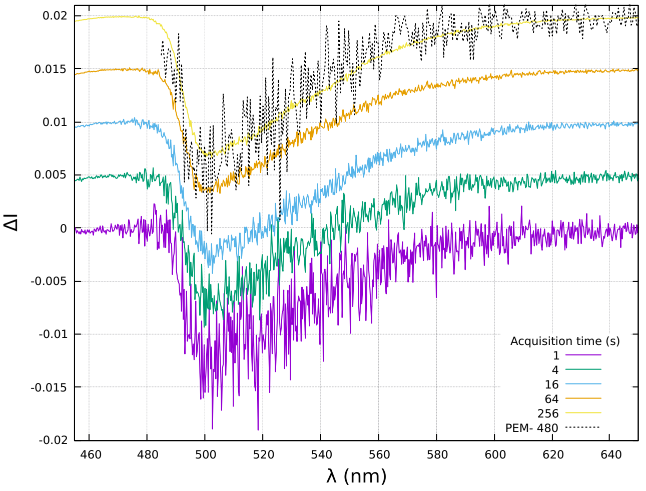

Supplementary Figure 7: CPL spectra of camphorequinone excited with a λ =450 nm laser, recorded with the CCD spatial-time configuration at different integration times from 1-256 s and the counterpart, in black-line, recorded with the step by step (PEM + analyzer) based setup, with 0.7 s/step integration time.

12 Mixing linear and PBS defects through fluorescein

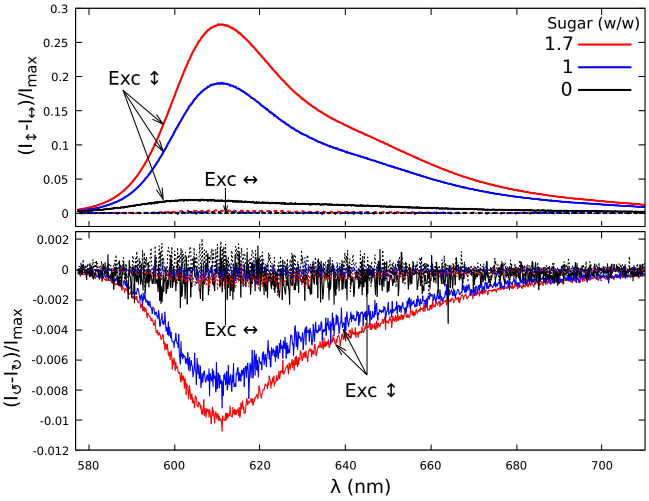

Supplementary Figure 8: Linear anisotropy (S_1) and CPL signal measured on a solution of fluorescein in water with different amount of sugar given in weight per weight. Fluorescence is detected at 90° to the excitation (450 nm LED). The excitation beam polarization is either vertical (continuous lines) or horizontal (dashed lines).

13 Chemical

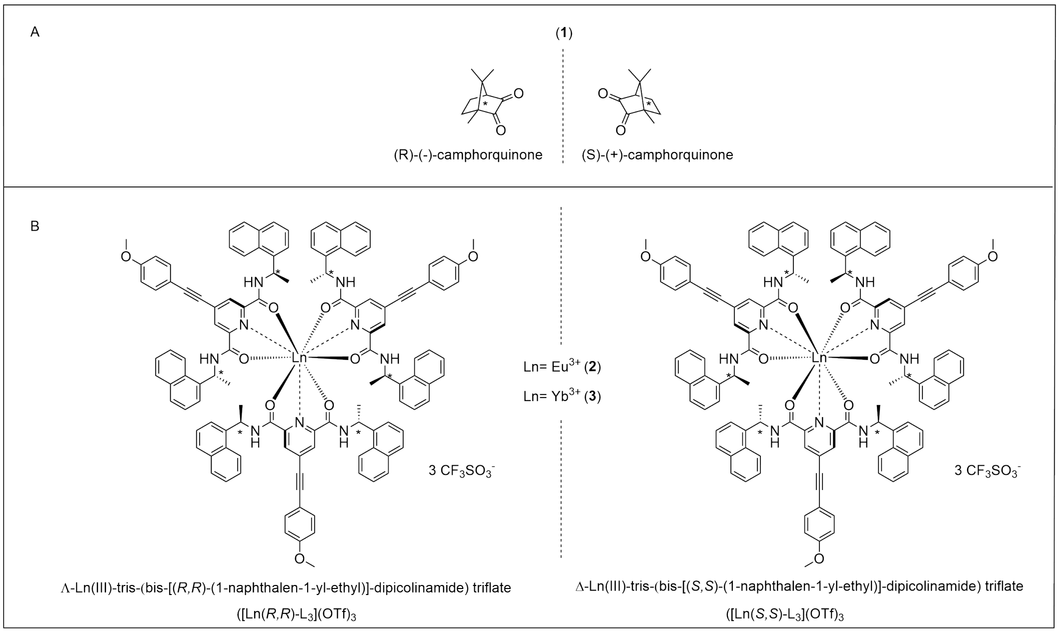

Supplementary Figure 9: Structures of chemical products.

14 Photoproperties

Molecules	λ_{abs}(nm)	ε ($M^{-1}cm^{-1}$)	λ_{em}(nm)	ϕ	$\lvert g_{lum} \rvert$	β	B_{CPL} ($M^{-1}cm^{-1}$)	τ_{int} (s)	REF
**1**	450 (Laser)	35	490	0,003	0,012	1	0,00063	20	[a]
**1**	270 (LED)	35	490	0,003	0,012	1	0,00063	100	
Eu^{3+}(**2**)	365	50000	594	0,3	0,2	0,05	75	0,1	[b]
		50000	597	0,3	0,1	0,11	82,5		
		50000	618	0,3	0,1	0,43	322,5		
		50000	622	0,3	0,05	0,26	97,5		
		50000	687	0,3	0,04	0,05	15		
		50000	694	0,3	0,04	0,09	27		
		50000	702	0,3	0,34	0,007	17,85		
Yb^{3+} (**3**)	365	50000	964	0,005	0,02	0,22	0,55	10	[b]
		50000	977	0,005	0,01	0,46	0,575		
		50000	988	0,005	0,12	0,3	4,5		
		50000	1013	0,005	0,04	0,02	0,1		
Heli	290	40000	1013	0,014	0,008	1	2,24	60	[c]

Supplementary Table 3: Photophysical parameters of 1 from ref [1], Eu^{3+} **2** and Yb^{3+} **3** from ref [2], with l_{exc} the excitation wavelength, e the molar absorption coefficient at the excitation wavelength, l_{em} the emission wavelength, f the quantum efficiency, $|g_{lum}|$ the dissymmetry factor absolute value with $g_{lum} = 2\frac{I_L - I_R}{I_L + I_R}$ where I_L and I_R are the left and right circularly polarized components of the luminescence, b the branching ratio, B_{CPL} the circularly polarized luminescence (CPL) brightness defined as $B_{CPL} = e \cdot b \cdot f \cdot |g_{lum}|/2$ and tint the integration time for the CPL measurements.

References

[1] A. Romani, G. Favaro, and F. Masetti. Luminescence properties of camphorquinone at room temperature. *Journal of Luminescence*, 63(4):183–188, mar 1995. 13

[2] Frédéric Gendron, Sebastiano Di Pietro, Laura Abad Galán, François Riobé, Virginie Placide, Laure Guy, Francesco Zinna, Lorenzo Di Bari, Amina Bensalah-Ledoux, Yannick Guyot, Guillaume Pilet, Fabrice Pointillart, Bruno Baguenard, Stephan Guy, Olivier Cador, Olivier Maury, and Boris Le Guennic. Luminescence, chiroptical, magnetic and ab initio crystal-field characterizations of an enantiopure helicoidal yb(iii) complex. *Inorg. Chem. Front.*, 8:914–926, 2021. 13
